# Phylogenetic and Ecologic Perspectives of a Monkeypox Outbreak, Southern Sudan, 2005

**DOI:** 10.3201/eid1902.121220

**Published:** 2013-02

**Authors:** Yoshinori Nakazawa, Ginny L. Emerson, Darin S. Carroll, Hui Zhao, Yu Li, Mary G. Reynolds, Kevin L. Karem, Victoria A. Olson, R. Ryan Lash, Whitni B. Davidson, Scott K. Smith, Rebecca S. Levine, Russell L. Regnery, Scott A. Sammons, Michael A. Frace, Elmangory M. Mutasim, Mubarak E. M. Karsani, Mohammed O. Muntasir, Alimagboul A. Babiker, Langova Opoka, Vipul Chowdhary, Inger K. Damon

**Affiliations:** Author affiliations**:** Centers for Disease Control and Prevention, Atlanta, Georgia, USA (Y. Nakazawa, G. L. Emerson, D.S. Carroll, H. Zhao, Y. Li, M.G. Reynolds, K. L. Karem, V. A. Olson, R. R. Lash, W. B. Davidson, S. K. Smith, R. S. Levine, R. L. Regnery, S. A. Sammons, M.A. Frace, I.K. Damon);; National Public Health Laboratory, Khartoum, Sudan (E.M. Mutasim, M.E.M. Karsani); Federal Ministry of Health, Khartoum (M.O. Muntasir, A.A. Babiker);; World Health Organization Regional Office for the Eastern Mediterranean, Cairo, Egypt (M.L. Opoka);; Médecins Sans Frontières, Khartoum (V. Chowdhary)

**Keywords:** Monkeypox, monkeypox virus, MPXV, viruses, phylogenetics, ecological niche modeling, Sudan, southern Sudan, South Sudan

## Abstract

Identification of human monkeypox cases during 2005 in southern Sudan (now South Sudan) raised several questions about the natural history of monkeypox virus (MPXV) in Africa. The outbreak area, characterized by seasonally dry riverine grasslands, is not identified as environmentally suitable for MPXV transmission. We examined possible origins of this outbreak by performing phylogenetic analysis of genome sequences of MPXV isolates from the outbreak in Sudan and from differing localities. We also compared the environmental suitability of study localities for monkeypox transmission. Phylogenetically, the viruses isolated from Sudan outbreak specimens belong to a clade identified in the Congo Basin. This finding, added to the political instability of the area during the time of the outbreak, supports the hypothesis of importation by infected animals or humans entering Sudan from the Congo Basin, and person-to-person transmission of virus, rather than transmission of indigenous virus from infected animals to humans.

Monkeypox is caused by a member of the genus *Orthopoxvirus*, first identified as the cause of disease in captive cynomolgus monkeys in 1959 (*1*). Twelve years later, the virus was identified as the cause of smallpox-like disease in humans (*2*). Although monkeypox virus (MPXV) can infect a wide variety of animal species when experimentally introduced, it is currently unknown which species are directly involved in its natural transmission cycle and whether >1 species are responsible for MPXV perpetuation in nature (*3*). Multiple events of human-to-human transmission have been reported, but sustained MPXV infection cycles among humans have not been documented (*4*–*6*). Likos et al. (*7*) investigated phylogenetic relationships between MPXV isolates by examining 5 whole-genome sequences. That analysis confirmed the existence of 2 distinct groups suggested by previous studies (*8*–*10*): the first group contained isolates from the Congo Basin (Congo Basin clade), and the second group included isolates from countries in western Africa. Differences in epidemiologic and clinical features between MPXV isolates (e.g., higher rates of illness and death of the Congo Basin clade) support the differentiation between these 2 clades.

In 2005, an outbreak of monkeypox among humans was reported from Unity State, Sudan (now South Sudan) (*4*); 19 cases were identified (*5*). Monkeypox cases among humans derived from contact with native animals have been reported in central and western Africa only; thus, this outbreak in Sudan could represent, if zoonotic transmission is confirmed, endemic transmission of monkeypox outside the recognized geographic range of the disease (*7*,*11*). Preliminary genetic and serologic analyses and epidemiologic investigations of the 2005 outbreak in Sudan showed ecological and genetic differences between the causative agent of this outbreak and of those that caused central and western African monkeypox outbreaks, and suggested that it could potentially be a novel virus (*5*). However, evidence indicating that the outbreak resulted from local virus transmission from wildlife to humans has not been presented.

Ecological niche modeling (ENM) has been used in the study of the ecological characteristics and distribution of a variety of diseases, such as dengue fever (*12*), leishmaniasis (*13*), plague (*14*,*15*), tularemia (*14*,*16*), West Nile virus infection (*17*), avian influenza (*18*,*19*), filovirus infections (*20*,*21*), and monkeypox (*22*–*24*). ENM is used as a tool for analyzing and identifying ecological requirements for the transmission of diseases and for localizing the geographic areas in which these requirements are met. When applied to human cases of monkeypox, ENM has enabled detection of an environmental signal common to all reported cases, which successfully predicts the range of the 2 recognized clades of monkeypox throughout the humid lowland forest regions of Africa (*23*). The area where the 2005 outbreak occurred represents a drier climate, and the dominant vegetation is substantially different from that in areas where monkeypox viruses from either of the 2 clades have been reported. Furthermore, Sudan has not been recognized as an area of potential favorability for MPXV transmission by previous ENM analyses.

To examine 2 hypotheses about the origin of the virus that caused the outbreak in Sudan, we explored genetic and ecological evidence from the 2005 Sudan outbreak and compared this evidence with what is currently understood about viruses in the 2 recognized clades of MPXV. The first hypothesis is that there was a previously unrecognized MPXV strain circulating naturally in the area of the outbreak; the second hypothesis is that the virus was imported into the area from a place where monkeypox is endemic. We used 2 independent lines of investigation: 1) the genetic characterization of the virus isolates from Sudan (Sudan isolates 1 and 2) and their comparison with previous isolates of MPXV from various regions of Africa by using phylogenetic analysis and 2) the generation of ecological niche models and characterization of ecological factors associated with monkeypox virus transmission on the basis of reported human cases in central and western Africa, including the assessment of environmental suitability for MPXV transmission among the Sudan localities.

## Materials

### Genetic Analysis

**Figure 1:**
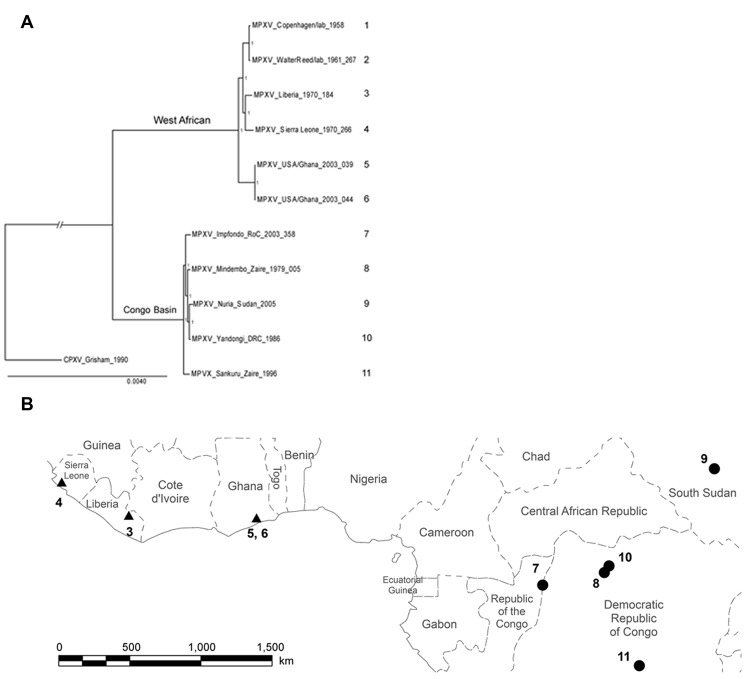
A) Phylogenetic tree produced from genome sequences (189,830 nt) of the 11 MPXV isolates. The separation between West African and Congo Basin clades is highly supported; the Sudan isolate is included within the Congo Basin clade. Posterior probabilities are indicated by the number 1 at each node. Scale bar indicates nucleotide substitutions per site. B) Map of geographic distribution of the isolates. Numbers correspond to those in Table 1; strains 1, Copenhagen 1958 and 2, Walter Reed 1961, were from laboratory samples and are not represented on the map. 3, Liberia 1970; 4, Sierra Leone 1970; 5, USA/Ghana 2003 039; 6, USA/Ghana 2003 044; 7, Impfondo 2003; 8, Mindembo 1979; 9, Nuria 2005; 10, Yandongi 1986; and 11, Sankuru 1996. Triangles indicate West African clade; circles indicate Congo Basin clade.

In addition to using the MPXV isolates used in the phylogenetic analysis by Likos et al. (*7*), we included 6 more isolates: 2 of these isolates correspond to strains that cause monkeypox outbreaks in laboratory animals (Copenhagen and Walter Reed) (*24*), 1 from Sierra Leone (*24*), 1 from Yandongi in the Democratic Republic of Congo (DRC), and 2 from the Sudan outbreak during 2005. The latter 2 isolates were found to be identical after alignment and were regarded as 1 genome, MPXV_Nuria_Sudan_2005, during our analysis (Table 1, Figure 1). In total, 11 isolates were used in the phylogenetic analysis.

**Table 1:** Virus isolates included in the phylogenetic analysis.of monkeypox outbreak, southern Sudan, 2005*

Isolate	Isolate no.	Source	GenBank accession no.	Clade
Copenhagen 1958	1	Cynomolgus monkey/laboratory	AY753185	West African
Walter Reed 1961	2	Cynomolgus monkey/laboratory	AY603973	West African
Liberia 1970	3	Human/wild	DQ011156	West African
Sierra Leone 1970	4	Human/wild	AY741551	West African
USA/Ghana 2003_039	5	Human/wild	DQ011157	West African
USA/Ghana 2003_044	6	Prairie dog/wild	DQ011153	West African
Impfondo RoC 2003	7	Human/wild	DQ011154	Congo Basin
Mindembo Zaire 1979	8	Human/wild	DQ011155	Congo Basin
Nuria Sudan 2005	9	Human/wild	KC257459	Congo Basin
Yandongi DRC 1986	10	Human/wild	KC257460	Congo Basin
Sankuru Zaire 1996	11	Human/wild	NC_003310	Congo Basin

*DRC, Democratic Republic of Congo.

### ENM

#### Human Case Data

**Figure 2:**
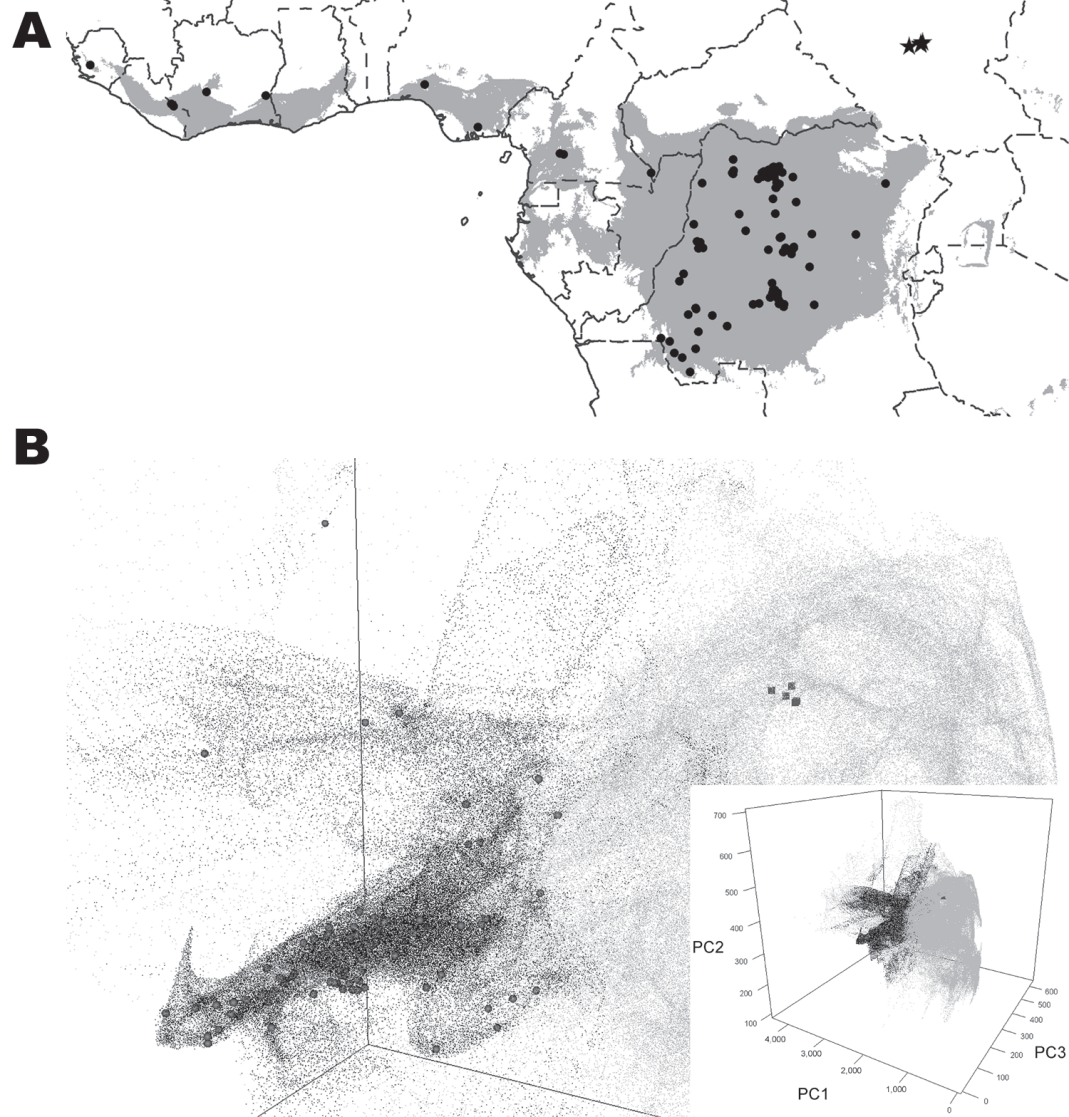
A) Predicted geographic distribution in central and western Africa of suitable environments for monkeypox virus transmission on the basis of the Maxent algorithm (www.cs.princeton.edu/~schapire/maxent/). Gray shading represents suitable environmental conditions identified by the algorithm; circles indicate localities of human monkeypox cases used to build the ecological niche models. Stars indicate localities reported during the human monkeypox outbreak in southern Sudan in 2005. B) Scatterplot using the first 3 principal components (PC1, PC2, and PC3) of the environmental variables in sub-Saharan Africa. Gray dots, environmental conditions in the entire area; black dots, suitable conditions identified by the ecological niche models; human monkeypox case localities; green squares, localities where monkeypox was reported in southern Sudan. Inset shows scatterplot scale.

We reviewed the reported human monkeypox cases in Africa, which were georeferenced at the patient’s residence village by using digital versions of 1:250,000 Joint Operational Graphic (www.map-reading.com/appendd.php) topographic maps from DRC and GEOnet Names Server (http://earth-info.nga.mil/gns.html/index.html) in tandem with detailed case information from the original reports, and following georeferencing procedures from MaNIS (*25*). Details of these procedures are provided in greater depth in a separate publication (*26*). The geographic coordinates of exposure locations for each case and its associated uncertainty were summarized in a database from which we selected all unique localities with the highest geographic confidence (small spatial uncertainty). Our final database contained 116 unique occurrence localities for Congo Basin and West African clades (Figure 2). Human monkeypox cases during the 2005 outbreak in Sudan were reported from 4 villages: Nuria, Bentiu, Rubkona, and Modin (Figure 1).

#### Environmental Datasets

We used 7 low-correlated bioclimatic variables from Worldclim (http://www.worldclim.org/) at a spatial resolution of 2.5 km to train the ecological niche models (*27*,*28*). These variables included annual mean temperature, mean diurnal range, maximum temperature of the warmest month, minimum temperature of the coldest month, annual precipitation, precipitation of the wettest month, and precipitation of the driest month.

## Methods

### Genetic Analysis

#### Sequencing and Alignment

**Figure 3:**
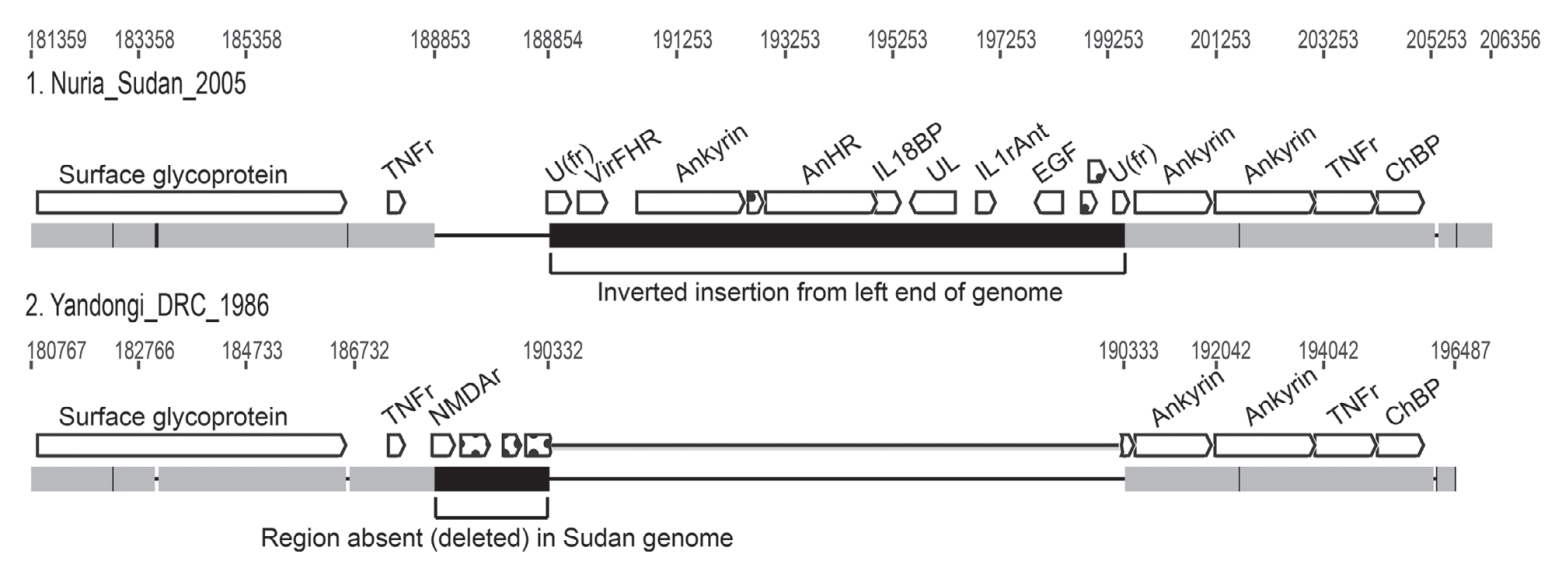
Comparison of a right-end segment from genomes of monkeypox virus (Nuria Sudan 2005 and Yandongi DRC1986. Numbers above genome map are nucleotide positions. Gray boxes represent DNA sequence identity in the 2 genomes; black represents differences. The 2 large black boxes illustrate the insertion/deletion event found in Sudan isolates 1 and 2. A region from the left end of the genome has been inserted where a portion of the right end (shown in Yandongi) has been deleted. Thin black horizontal lines represent gaps in the alignment. Open reading frames are shown in white. Open reading frame names were assigned with reference to MPXV genomes available at the Poxvirus Bioinformatics Resource Center (www.poxvirus.org). Segment boxes with dots indicate unknown genome sequences; TNFr, tumor necrosis factor receptor; U(fr), unknown fragment; VirFHR, virulence factor host range; AnHR, ankyrin host range; IL18BP, interleukin 18 binding protein; UL, ubiquitin ligase; IL1rAnt interleukin 1 receptor antagonist; EGF, epidermal growth factor; ChBP, chemokine binding protein; NMDAr, *N*-methyl D-aspartate receptor-like protein.

We used previously described Sanger sequencing methods to sequence the genomes of 2 MPXV isolates collected in southern Sudan (MPXV_Nuria_Sudan_2005, 1 and 2) and an isolate from northern DRC (MPXV_Yandongi_DRC_1986), isolated from a scab collected from an 8-month-old boy. An alignment was created from complete genome sequences from 11 MPXV isolates and cowpox virus Grisham (CPXV_GRI) by using MAFFT version 6 (http://mafft.cbrc.jp/alignment/server/) (*29*). All columns containing gaps were then removed. Consequently, the insertion/deletion region (bp 188854–199543) in MPXV_Nuria_Sudan_2005 sequence from Sudan isolates 1 and 2 was removed (Figure 3). The final alignment was 189,830 bp and was identical in the 2 Sudan isolates; thus, only 1 isolate was used in the analyses.

### Phylogenetic Analysis

A maximum clade credibility tree was generated from the sequence matrix by using MrBayes (*30*,*31*) under the general time reversible + proportion invariant + Γ model with the following settings: lset nst = 6, rates = invgamma, mcmc ngen 5,000,000, sample freq = 1,000, nchains = 4, startingtree = random, burnin = 500. The model was chosen because it allows for variable base frequencies, rate variation among sites, and a proportion of invariable sites within the matrix. No priors were specified because the default priors are expected to work well for most analyses and parameters are then estimated from the data assuming no prior knowledge of their values. The final average standard deviation of split frequencies for the 2 runs was 0.00000, demonstrating convergence.

### ENM Procedures

We used 2 algorithms, Genetic Algorithm for Rule-Set Production (GARP [http://openmodeller.sourceforge.net/index.php?option=com_content&task=view&id=8&itemid=4]) and Maxent (www.cs.princeton.edu/~schapire/maxent/), to create ecological niche models of monkeypox transmission by using localities where the 2 recognized clades (Congo Basin and West African) were identified in human samples. GARP is based on a genetic algorithm and is used to find a set of rules describing nonrandom associations between localities where disease transmission has been reported and environmental conditions in those localities (*32*,*33*). These rules are built and selected through an iterative process of creation, evaluation, modification, and inclusion or exclusion of rules that follow 4 basic forms (bioclimatic, atomic, negated, and logistic regression); this process stops when a maximum number of iterations (1,000) is met or an optimization parameter changes by <1% from 1 generation to the next. We tested 500 models by using Desktop GARP (www.nhm.ku.edu/desktopgarp/index.html). We used 50% of points for training the model and 50% to test it. We then selected 50 models by using the best subset option (20% of soft omission threshold distribution and 50% of commission threshold distribution); all other parameters were left as default values. The 50 selected models were then combined. The predicted area was defined as a combination of those areas with higher or equal model coincidence value than the lowest model coincidence value for the human case-patient localities.

Maxent is used to estimate a probability distribution by comparing the environmental conditions at localities in which disease transmission was recorded with environmental conditions across the landscape (*34*,*35*). We used the maximum entropy principle calculation of the program to find this estimated distribution; thus, the result was the closest to a uniform distribution with a mean that was closest to the observed mean value from known occurrences, achieved by a regularization parameter (β). We used the default values for all parameters; the predicted area was then selected by using a lowest probability threshold value to differentiate between suitable and nonsuitable areas (*36*).

### Principal Component Analysis

By using the 7 environmental variables described, we performed a principal component analysis to describe the environmental variability in sub-Saharan Africa and identify those conditions in which monkeypox cases in humans have been recorded and those conditions identified as suitable by ENM analysis. We used the principal components tool in ArcGIS 10 (Esri, Redlands, CA, USA) to calculate the principal components and used the rgl package 0.92.798 (http://ftp.osuosl.org/pub/cran/) for R 2.13.1 (*37*) to visualize the environmental conditions at the localities where monkeypox had been reported, in the areas predicted by ENM algorithms to be suitable for monkeypox transmission, and in the localities of the Sudan outbreak in 2005 in association with the conditions in sub-Saharan Africa. Additionally, we calculated basic statistical parameters (mean, maximum, minimum, and SD) for each variable within the area predicted by the ENM algorithms, which, in turn, were compared with the environmental conditions found at the locations proposed as transmission sites in Sudan.

## Results

### Genetic Analysis

We used a maximum clade credibility tree, which displayed high support at all nodes (Figure 1). Six isolates were grouped within the clade identified in western Africa: Liberia, Sierra Leone, 2 isolates from Ghana, and 2 isolates acquired from primate colony outbreaks and subsequently extensively passaged in cell culture (Copenhagen and Walter Reed). The remaining 5 isolates were grouped within the Congo Basin clade, including the 2 isolates obtained during the Sudan outbreak in 2005 that were processed and combined for analysis. The isolate from Sudan was most closely related to isolates from Yandongi and Mindembo, located in north central DRC. Of isolates examined, these were from locations nearest to Sudan (Figure 2). 

Figure 3 shows a genotypic map of a unique indel region in the right side of the MPXV_Nuria_Sudan_2005 genome that represents a large inverted duplication originating from the left end. This unusual (10.8 kbp) duplication found in Sudan isolates 1 and 2 is composed of open reading frames of several host immune modulator genes (MPXV_Zaire_1979–005 open reading frames 5–16) and some fragments of the inverted terminal repeats. This duplication was partially lost in 1 of the 2 isolates after the second BSC40 tissue culture passage. Additional sequence variations between Sudan isolates 1 and 2 occurred at 4 locations (these variations were also excluded from the phylogenetic analysis because they created gaps that were removed during the alignment): at nt position 10838, Sudan isolate 2 has additional copies of a repeat TTAGA (this variation is in the inverted terminal repeat and so is also reflected at the right end of the genome); at nt 20935, Sudan isolate1 has an additional T in a homopolymer string; at nt 138827, Sudan isolate 1 has an additional ATC repeat; at nt 179133, Sudan isolate 1 has a repeat of 23[ATATACATT] not present in Sudan 2. One of these 4 variations occurs in a coding region. The additional ATC repeat is found in the P4c precursor gene where it codes for an additional aspartic acid residue. The complete genome sequence, absent the full inverted terminal repeat regions and hairpin ends, of the Sudan 1 isolate was 206,346 nt.

A comparison of the 189,830-nt alignment data (Table 2) revealed no nt differences between Sudan isolates 1 and 2, 21 nt differences between MPXV_Nuria_Sudan 2005 and MPXV_Yandongi_DRC_1986, and 34 nt differences between Sudan and Mindembo. Notably, isolates from Yandongi and Mindembo were collected in 1986 and 1979, respectively (19 and 26 years before the monkeypox outbreak in Sudan), and there are 34 nt differences between these 2 isolates. We compared data from the monkeypox outbreaks in Yandongi, Sankuru, and Impfondo and found 55 nt differences between isolates from Yandongi and Sankuru and 44 between those from Yandongi and Impfondo; locations in these pairs are separated by a geographic distance less than that between Sudan and Yandongi. Consequently, the genetic distance between Sudan and Yandongi isolates is low in comparison with other pairs, especially considering the greater number of years between collection dates of the 2 isolates and greater geographic distance.

**Table 2:** Nucleotide differences and distances between genome sequences (189,830 nt) of monkeypox virus isolates, 1958–2005*

Isolate	Copenhagen 1958	Liberia 1970	Sierra Leone 1970	Walter Reed 1961	USA/ Ghana 039 2003	Sankuru Zaire 1996	Impfondo RoC 2003	Mindembo Zaire 1979	Yandongi DRC 1986	Sudan 2005
Copenhagen 1958	–									
Liberia 1970	74	–								
Sierra Leone 1970	81	67	–							
Walter Reed 1961	7	73	80	–						
USA/Ghana 039 2003	130	138	143	129	–					
Sankuru Zaire 1996	1,000	1,007	1,017	1,001	1,029	–				
Impfondo RoC 2003	1,005	1,012	1,022	1,006	1,034	59	–			
Mindembo Zaire 1979	998	1,005	1,015	999	1,027	56	43	–		
Yandongi DRC 1986	1,003	1,009	1,020	1,004	1,032	55	44	34	–	
Sudan 2005	1,012	1,018	1,029	1,013	1,041	66	55	34	21	–

*Dashes indicate 100% identity points; blank cells indicate identical values to those below the diagonal; shaded cells indicate within-clade variation; raw number of nucleotide differences between pairs of isolates. DRC, Democratic Republic of Congo.

### Ecological Niche Models

The areas with suitable environmental conditions obtained from the GARP and Maxent models were similar; thus, we present only the results from Maxent (Figure 2). We used Maxent probability >0.0903047 and GARP model coincidence ≥34 to determine the areas predicted by the model because these are the lowest probability and coincidence values at the localities used to train the models (i.e., omission error = 0). Reported cases from southern Sudan do not fall within the suitable areas predicted by the ENM algorithms (Figure 2).

Table 3 summarizes the environmental conditions found in the areas predicted as suitable by the ecological niche models for all 7 variables. In general, monkeypox cases in humans in Sudan were reported from areas with higher mean temperatures, lower annual precipitation, and higher temperature ranges than those from areas with indigenous monkeypox occurence. Annual precipitation values for the localities in Sudan are lower than suitable values predicted by Maxent and are at the drier end of values predicted by GARP; furthermore, values for precipitation of the driest month for Sudan localities reach 0 mm, although neither algorithm predicts such conditions to be suitable for MPXV transmission. Annual mean temperatures of Sudan localities are higher than mean values from ENM algorithm predictions but slightly lower than the maximum values from the models. Maximum temperature of the warmest month is consistently higher in Sudan than in either ENM prediction.

**Table 3:** Ecological analyses of monkeypox outbreak, Southern Sudan, 2005*

Locality	Value	AMT, °C	MDR, °C	MTWM, °C	MTCM, °C	AP, mm	PWM, mm	PDM, mm
Sub-Saharan Africa	Mean	23.60	13.052	33.309	13.060	978.17	195.34	9.23
	Min	−3.0	2.7	3.5	−8.6	8	3	0
	Max	31.9	20.8	42.5	23.3	4552	1157	165
	SD	3.525	2.459	3.933	5.589	569.75	97.94	18.54
Sudan	Mean	27.4	13.925	37.3	17.525	821	193.25	0
	Min	27.3	13.9	37.1	17.5	815	192	0
	Max	27.6	14.0	37.7	17.6	829	195	0
	SD	0.141	0.05	0.282	0.05	5.83	1.25	0
SPA GARP	Mean	24.34	10.59	31.36	17.55	1,680.45	253.6	32.86
	Min	16.1	7.4	24.5	5.9	803	135	3
	Max	27.80	15.4	36.9	21.9	2902	699	137
	SD	1.392	1.156	1.63	2.170	263.133	54.746	28.442
SPA Maxent	Mean	24.74	10.24	31.32	18.43	1,745.54	251.6	38.26
	Min	13.7	5.4	19.4	7.8	941	157	1
	Max	27.8	14.5	37.3	23.0	3303	798	137
	SD	1.224	1.085	1.41	1.927	254.025	60.917	29.946

*Deviation values for climatic environmental variables within the study region (sub-Saharan Africa); at georeferenced points of human monkeypox occurrences in Sudan; and within the suitable predicted areas for monkeypox identified by each algorithm, GARP, Genetic Algorithm for Rule-Set Production (http://openmodeller.sourceforge.net/index.php?option=com_content&task=view&id=8&itemid=4) and Maxent [www.cs.princeton.edu/~schapire/maxent/). AMT, annual mean temperature; MDR, mean diurnal range; MTWM; maximum temperature of the warmest month; MTCM, minimum temperature of the coldest month; AP, annual precipitation; PWM, precipitation of the wettest month; PDM, precipitation of the driest month; Min, minimum; Max, maximum; SPA, suitable predicted areas for monkeypox transmission.

The first 3 components describe >99% of the environmental variability in sub-Saharan Africa on the basis of the 7 selected variables (principle component [PC]1 = 97.47%, PC2 = 1.35% and PC3 = 0.84%). Figure 2 shows the distribution within sub-Saharan environments, historic MPXV case localities, and localities corresponding to the 2005 Sudan monkeypox outbreak. The latter localities fall outside the suitable environmental conditions for MPXV transmission predicted by ENMs.

## Discussion

Our phylogenetic analysis strongly supports the existence of distinct clades from the Congo Basin and western African, and all subclades were well supported. Sudan isolates 1 and 2 are imbedded within the Congo Basin MPXV clade, specifically within a northern DRC subclade. However, we did not have samples from the northernmost Congo Basin forest, which is closer to Sudan. In comparison of the position of the isolates from the 2005 Sudan outbreak with that of other isolates from Congo Basin during 1986–2003, the former cannot be distinguished as a new strain of MPXV on the basis of these phylogenetic analyses. Furthermore, the isolates most closely related to the Sudan isolate are from Yandongi and Mindembo DRC, suggesting that the virus obtained during the 2005 outbreak probably originated from northern DRC (Figure 1).

The Sudan isolates uniquely duplicate a 10.8-kb sequence that represents a single mutation event. Given the overall similarity to the Congo Basin isolates, this single duplication is not considered sufficient evidence to suggest an independent evolutionary trajectory. Formenty et al. (*5*) proposed that the Sudan virus was novel among Congo Basin isolates because of this large duplication of genetic information not seen in other monkeypox viruses sequenced to date. The changes seen in the 4 regions between the 2 sequenced Sudan isolates were not seen in the sequenced monkeypox isolates from the 2003 US outbreak of monkeypox. Further genetic analyses could help clarify epidemiologic details through examination of genetic variations accumulated during a single outbreak, but these analyses are beyond the scope of the current study.

The long-term maintenance and transmission of a virus in wildlife would presumably require genotypic adaptations to susceptible hosts, which in turn are adapted to the environmental characteristics of a particular region. Although there are some differences between the Sudan MPXV and other Congo Basin viruses, these differences are well within the limits of variation seen within the Congo Basin clade. The 2 recognized MPXV clades (West Africa and Congo Basin clades) have been described in areas in which the dominant ecosystem is tropical rainforest. Although there are slight habitat differences between the MPXV ranges within western Africa and the Congo Basin, the grassland environmental characteristics and habitat descriptions at the outbreak localities in Sudan are dramatically different and do not fit the expected suitable environmental conditions on the basis of current knowledge of the 2 MPXV clades. This observation is supported by the ENMs (Figure 2), in which the 2005 outbreak localities are not identified as suitable for MPXV transmission and life cycle maintenance.

The 2 possible explanations for the source of the virus that caused the 2005 monkeypox outbreak in Sudan are 1) the existence of conditions permitting the long-term maintenance of MPXV in wildlife within the area where this outbreak occurred and transmission of the virus from reservoir hosts into humans; and 2) the importation of MPXV into the outbreak area by an infected human or animal. The first hypothesis cannot be supported by the results from ENMs and the criteria of ecological niche conservatism between genetically differentiated taxa, which demonstrate that genetic differentiation occurs faster than ecological differentiation (*38*). On the basis of the ecological differences described in this study, we would expect indigenous isolates from Sudan to have high genetic differentiation when compared with isolates from MPXV clades found in West Africa and Congo Basin. Our genetic analysis, however, groups Sudan isolates 1 and 2 within the Congo basin clade; therefore, we consider the hypothesis of an indigenously acquired infection to be unlikely.

Genetic similarity between isolates from DRC and the sample obtained from the 2005 Sudan MPXV outbreak support the second hypothesis, importation of the virus. Additionally, human nomadic events, displacement and repatriation of Sudanese residents, were characteristic of southern Sudan and northeastern DRC during the time of the outbreak (*39*). Members of the Lord’s Resistance Army fled from Sudan into the Oriental Province of DRC persecuted by the Uganda People’s Defense Forces during September 2005 and were reported to have left the area by October (http://www.irinnews.org/report.aspx?reportid = 57083). In January of that year, the Comprehensive Peace Agreement was signed, representing the end of a civil war that started in 1983 between northern and southern Sudan and giving autonomy to southern Sudan until 2011, when a referendum on independence was held, resulting in the recognition of South Sudan as a country. The end of the civil strife in Sudan precipitated the return of refugees who had sought assistance in neighboring countries (including DRC). Some sources reported the spontaneous return of thousands of persons from southern Sudan to their homeland in 2005 (http://reliefweb.int/node/198511). Whether the movements of these persons are linked to the cases of monkeypox in Sudan may never be known with certainty, but the circumstances could have facilitated the importation of the disease by translocation of an infected animal or person from DRC.

An MPXV endemic to Sudan should reflect its adaptation to different hosts and ecological environments with respect to the currently known areas where the disease is endemic in the form of genetic divergence. Given the dramatic difference in ecology between the region surrounding Nuria, Sudan, and historic points of MPXV occurrence, the genomic comparisons between the Sudan isolate and other strains of MPXV would be expected to reveal genetic divergence as great as or even exceeding that observed between the 2 currently recognized MPXV clades in western and central Africa. However, our data indicate that the Sudan MPXV isolates and an MPXV isolate from Yandongi in north-central DRC are genetically similar to each other, even though they were collected 19 years apart from ecologically disparate and geographically discrete locations. For reference, these isolates from Yandongi and Sudan were more genetically similar to each other than the Yandongi and Mindembo DRC isolates (Figure 1), that were the geographically and temporally closest isolates studied.

Further serologic surveys of human, animal, or both populations in Sudan could provide useful evidence in the investigations of the origin of the virus that caused this outbreak in Sudan. In addition, increased disease surveillance, ecological studies, and further characterization of the variability within and between clades will improve our understanding of the natural history of MPXV. Further epidemiologic studies to identify the sources and potential risks of MPXV infection in localities inside and outside the areas in which the disease is known to occur are clearly warranted.
